# A Golgi-Localized Sodium/Hydrogen Exchanger Positively Regulates Salt Tolerance by Maintaining Higher K^+^/Na^+^ Ratio in Soybean

**DOI:** 10.3389/fpls.2021.638340

**Published:** 2021-03-09

**Authors:** Tianjie Sun, Nan Ma, Caiqing Wang, Huifen Fan, Mengxuan Wang, Jie Zhang, Jinfeng Cao, Dongmei Wang

**Affiliations:** ^1^State Key Laboratory of North China Crop Improvement and Regulation, Baoding, China; ^2^Key Laboratory of Hebei Province for Plant Physiology and Molecular Pathology, Baoding, China; ^3^College of Life Sciences, Hebei Agricultural University, Baoding, China; ^4^Hebei Key Laboratory of Crop Salt-Alkali Stress Tolerance Evaluation and Genetic Improvement, Cangzhou, China; ^5^Academy of Agricultural and Forestry Sciences, Cangzhou, China

**Keywords:** salt stress, VIGS, *GmNHX5*, CRISPR/Cas9, hairy roots

## Abstract

Salt stress caused by soil salinization, is one of the main factors that reduce soybean yield and quality. A large number of genes have been found to be involved in the regulation of salt tolerance. In this study, we characterized a soybean sodium/hydrogen exchanger gene *GmNHX5* and revealed its functional mechanism involved in the salt tolerance process in soybean. *GmNHX5* responded to salt stress at the transcription level in the salt stress-tolerant soybean plants, but not significantly changed in the salt-sensitive ones. GmNHX5 was located in the Golgi apparatus, and distributed in new leaves and vascular, and was induced by salt treatment. Overexpression of *GmNHX5* improved the salt tolerance of hairy roots induced by soybean cotyledons, while the opposite was observed when *GmNHX5* was knockout by CRISPR/Cas9. Soybean seedlings overexpressing *GmNHX5* also showed an increased expression of *GmSOS1*, *GmSKOR*, and *GmHKT1*, higher K^+^/Na^+^ ratio, and higher viability when exposed to salt stress. Our findings provide an effective candidate gene for the cultivation of salt-tolerant germplasm resources and new clues for further understanding of the salt-tolerance mechanism in plants.

## Introduction

Soybean is one of the most widely cultivated oil crops worldwide. The usage of soybean products includes human foods, animal foods, industrial products, ingredients, precursor materials, etc. (Gaonkar and Rosentrater, 2019). The production of soybean is restricted by a variety of adverse factors, among which salt stress caused by soil salinization is one of the most serious factors that harm soybean yield and quality (Hasanuzzaman et al., 2016). Salt stress impairs soybean growth in many ways. On the one hand, salt stress inhibits the formation of nodules to weaken the assimilation of nitrogen (Singleton and Bohlool, 1984); on the other hand, high osmotic pressure causes cells to lose water and suffer toxic effects (Massa and Melito, 2019). Salt stress disrupts the dynamic equilibrium established by the reactive oxygen species (ROS)-scavenging system (Moradi and Ismail, 2007; Esfandiari and Gohari, 2017), causing the increase of membrane permeability and malondialdehyde (MDA) content due to the oxidation of membrane lipids (Geilfus, 2019).

Plants have evolved multiple ways to enhance their adaptability under salt stress. Cells selectively absorb salt and compartmentalize salt into vacuoles to maintain a relatively stable internal environment, or transport excess salt to older tissues to avoid damage to the young tissues that are more sensitive to salt stress (Hines, 2008; Wang et al., 2019). The inorganic ions absorbed by plants mainly comprise Na^+^, K^+^, Ca^2+^, Cl^–^, among which the balance of intracellular K^+^/Na^+^ concentration is the key to ensure normal physiological metabolism (Maathuis and Amtmann, 1999; James et al., 2011). In addition to the regulation of ion levels, plants also accumulate a series of osmotic adjustment substances, such as soluble saccharide, proline, and betaine (Qi et al., 2014), to regulate the osmotic potential and prevent the damage caused by ROS (Bohnert and Jensen, 1996).

Salt tolerance is an important trait in soybean breeding, but there have not been many salt-tolerant genetic resources available for breeding, which limits the development of new salt-tolerant soybean varieties. Therefore, it is of great significance to explore the molecular basis of salt tolerance in plants and to develop genetic resources that are available for genetic breeding. Among the molecular pathways that are involved in salt stress response, including protein kinases such as CIPK (Weinl and Kudla, 2009) and CDPK (Boudsocq and Sheen, 2012); transcription factors such as NAC (Tran et al., 2004), WRKY (Jiang and Deyholos, 2008), bHLH (Jiang et al., 2009), SERF (Schmidt et al., 2013), bZIP (Yang et al., 2009), and MYB (Yoo et al., 2005); channel proteins such as HKT (Horie et al., 2007) and AKT (Wang P. et al., 2015); ion pumps such as HAK (Wang et al., 2018), and ion exchangers such as NHX (Aviv, 1993) and SOS (Qiu et al., 2002), have been found to respond to salt stress and participate in the regulation of plant salt tolerance. The functions of NHX (Na^+^/H^+^ exchanger) family members have attracted growing attention of physiology researchers.

NHXs are monovalent ion exchangers that localize in the membrane, which catalyze the movement of Na^+^ or K^+^ (Barragán et al., 2012) to the side with high ion concentration, and at the same time exchange with an H^+^ across the membrane to maintain local potential conservation (Rodríguez-Rosales et al., 2009). Such functions maintain the ion concentration of cells (or organelles) on the one hand, and regulate the stability of the pH environment of the chamber on the other hand (Gadsby, 2009). In *Arabidopsis*, the NHX family consists of eight members, which can be divided into three categories according to their subcellular distribution: plasma membrane NHXs (AtNHX7/SOS1 and AtNHX8), vacuolar NHXs (AtNHX1-4), and inner membrane NHXs (AtNHX5 and AtNHX6) (Qiu, 2016). Up to now, most of the studies on the function of NHX family genes and their involvement in signaling pathways have focused on vacuolar membrane NHXs and plasma membrane NHXs. At the histological level, the expression of *SOS1* has been detected in epidermal cells at the root tip and in parenchyma cells at the xylem/symplast boundary of roots, stems, and leaves, controlling Na^+^ transport through the system of xylem system (Shi et al., 2002). At the cellular level, AtNHX1 transports Na^+^ (Pehlivan et al., 2016) or K^+^ (Barragán et al., 2012) to the vacuole in exchange for H^+^ to the cytoplasm, while SOS1 exports Na^+^ to Pehlivan et al. (2016), or import K^+^ from Wu et al. (1996), the extracellular space, thereby increase the K^+^/Na^+^ ratio in the cytoplasm. Our previous studies also demonstrated that heterologous expression of *GmNHX1* (a soybean NHX that localizes in the vacuole membrane) in *Arabidopsis* increased the flow rate of Na^+^ in the roots by up-regulating the expression of *SKOR*, *SOS1*, and *AKT1* genes under salt stress, thereby improve salt tolerance via increasing the K^+^/Na^+^ ratio (Sun et al., 2019).

To our knowledge, however, research on the NHXs distributed in the endomembrane system is rare. AtNHX5 and AtNHX6 are located in the *trans*-Golgi network and pre-vacuolar compartments, affecting the cargo sorting behavior of the *trans*-Golgi network by adjusting the pH in the vesicles (Reguera et al., 2015). AtNHX5 and AtNHX6 also maintain the steady-state of K^+^ and pH, allowing cells to accumulate higher K^+^ under acidic conditions (Wang L. et al., 2015). The *nhx5 nhx6* double mutant showed intense sensitivity to salt stress (Bassil et al., 2011a), and the heterologous expression of *AtNHX5* in *Broussonetia papyrifera* increases the tolerance to salt stress and drought (Li et al., 2011). It is worth noting that the molecular mechanism by which these NHXs related to the inner membrane system and participate in plant salt tolerance has not yet been fully revealed.

In this study, we characterize GmNHX5, a Golgi apparatus-localized NHX in soybean, and reveal the mechanism by which it participates in salt stress adaption in plants. The transcription level of *GmNHX5* increases significantly upon salt treatment in salt-tolerant soybean cultivars, whereas remains unchanged in salt-sensitive ones. The activity of the *GmNHX5* promoter is high in new leaves and vascular tissues and increases when exposed to salt stress. Hairy roots overexpressing *GmNHX5* show stronger salt tolerance, while the *GmNHX5* knocked-out hairy roots are more sensitive to salt stress than wild type. We further overexpress *GmNHX5* in soybean and find that the transformed plants show higher osmotic adjustment substances accumulation, K^+^/Na^+^ ratio, stronger salt tolerance, and higher expression levels of *GmSOS1*, *GmSKOR*, and *GmHKT1* under salt stress than wild type. Our findings provide a candidate gene for genetic breeding and offer clues to the molecular mechanism by which endometrial NHXs participate in the response to salt stress.

## Materials and Methods

### Plant Cultivation

Soybean cultivar Jidou-7 was obtained from the Institute of Grain and Oil Crops, Hebei Academy of Agricultural and Forestry Sciences, and cultivar Mustang was obtained from Plant Transformation Core Facility, University of Missouri. Soybean plants were cultivated in a greenhouse with a 14 h light/10 h dark cycle at a constant temperature of 25°C and 700 μmol photons m^–2^ s^–1^. Soybeans hairy roots were cultured in sterile petri dishes in a tissue culture room with the same light and temperature conditions as the greenhouse.

### Isolation of Plant RNA and Detection of Gene Expression

Total RNA was isolated using an RNA extraction kit (UNlQ-10 Column Trizol Total RNA Isolation Kit, Sangon Biotech), then reverse transcribed into cDNA through a reverse transcription kit (PrimeScript RT reagent Kit with gDNA Eraser, TaKaRa). RT-PCR and RT-qPCR experiments were performed using primers listed in Supplementary Table 1. *Actin* was used as a reference gene in the RT-PCR and RT-qPCR experiments according to our previous report (Ma et al., 2013). Three biological replicates with their respective three technical replicates were conducted for each sample in the RT-qPCR experiments and calculated using the 2^–ΔΔ*CT*^ method.

### Phylogenetic Analysis

Protein sequences were retrieved from UniProtKB through the accessions in Supplementary Table 2 before aligned using ClustalW (Thompson et al., 1994). The similarity between proteins was inferred by using the Maximum Likelihood method and JTT matrix-based model (Jones et al., 1992). The initial tree for the heuristic search was obtained automatically by applying Neighbor-Join and BioNJ algorithms to a matrix of pairwise distances estimated using a JTT model and then selecting the topology with superior log likelihood value. The tree was drawn to scale, with branch lengths measured in the number of substitutions per site. Evolutionary analyses were conducted in MEGA X (Kumar et al., 2018). The proteins in the phylogenetic tree were further colored based on their subcellular distribution analyzed based on their Gene Ontology (GO) terms (Gaudet et al., 2011).

### Plasmid Construction

In order to construct a plasmid for histological analysis of GmNHX5, purified DNA from soybean variety Jidou-7 was used as a template, and primer 1381-GmNHX5p-F/R was used to amplify the 2000 bp sequence upstream of the *GmNHX5* start codon. The amplified fragment was inserted between *Eco*RI and *Hin*dIII in plasmid pCAMBIA1381.

For the construction of the plasmid for GmNHX5 subcellular localization, primers GmNHX5GFP-F/R were used to amplify the CDS region of the *GmNHX5* gene using the cDNA of soybean cultivar Jidou-7 as a template, and the amplified fragment was inserted into the plasmid pCAMBIA3301-GFP between *Nco*I and *Eco*O65I.

The plasmid used to silence *GmNHX5* by VIGS was constructed by inserting a specific fragment of *GmNHX5* amplified using the primers GmNHX5VIGS-F/R and inserted into the pTRV2 plasmid between *Eco*RI and *Xho*I.

For the construction of the *GmNHX5*-overexpressing plasmid, the primers GmNHX5OE-F/R were used to amplify the CDS region of the *GmNHX5* gene using the cDNA of soybean cultivar Jidou-7 as a template, and the amplified fragment was inserted into the plasmid pCAMBIA3301 between *Nco*I and *Eco*O65I.

Targeted gene knockout was performed using the CRISPR/Cas9-based approach. The gene sequence of *GmNHX5* with introns was aligned with the whole soybean genome (Wm82.a2.v1, Phytozome) (Schmutz et al., 2010), and the appropriate sgRNA was designed according to a previously published protocol (Xie et al., 2014). Two specific sgRNAs (sgRNA-1: 5′-CAGACACCGAGACTAATATC-3′; sgRNA-2: 5′-ACTCCTTTAGTACTCAGTCT-3′) were used for *GmNHX5* knockout, according to the previously published method (Xing et al., 2014). To construct the *GmNHX5* knockout plasmid, the primers GmNHX5-DT1-BsF/F0/R0/BsR were used to amplify the plasmid pCBC-DT1T2 and inserted the amplified fragment into the Cas9 expression vector pBSE401 according to a previously reported protocol (Xing et al., 2014). All primers are listed in Supplementary Table 1.

### Detection of *GmNHX5* Histological Distribution

The histological distribution of *GmNHX5* promoter activity was detected by GUS staining. The T_2_ progeny of 5-day-old *Arabidopsis* seedlings transformed with *GmNHX5p*:GUS plasmid were transferred to MS medium containing 0, 100, or 200 mM NaCl, respectively, for 24 h. Histochemical GUS staining assays were performed using a GUS histochemical assay kit (Real-Times) following the manufacturer’s protocol. The seedlings were washed with 70% ethanol to dissolve chlorophyll and GUS staining was examined under a fluorescence microscope (BX53, Olympus). These experiments were repeated three times to ensure accuracy.

### Detection of GmNHX5 Subcellular Localization

The subcellular localization of GmNHX5 was observed using laser scanning confocal microscopy. The T_2_ progeny of *Arabidopsis* seedlings transformed with plasmids carrying *CaMV 35S*:GmNHX5-GFP or *CaMV 35S*:GFP expression loci were observed under a laser scanning confocal microscope (FV1000, Olympus). Fluorochrome Golgi-Tracker Red (Beyotime) was applied to the roots of 5-day-old *Arabidopsis* seedlings to label the Golgi apparatus following the manufacturer’s protocol. Fluorescence of GFP was excited at 488 nm and detected at 520–540 nm. Fluorescence of Golgi-Tracker Red was excited at 589 nm and detected at 605–635 nm. Line profiling was performed with Fiji (ImageJ^1^), in accordance with a previous study (Li et al., 2012). These experiments were repeated three times to ensure accuracy.

### Generation of *GmNHX5*-Silencing Plants

TRV-mediated VIGS was used to generate *GmNHX5*-silencing plants. The VIGS experiment was conducted according to a previously reported protocol (Muthappa and Mysore, 2014), with minor modifications. One-week-old soybean seedlings were used in the VIGS experiment. The empty plasmid pTRV2 was used as control (TRV: *00*). *Agrobacterium tumefaciens* carrying pTRV1 and recombinant pTRV2 plasmids were resuspended in the infection buffer [50 mM MES, 2 mM Na_3_PO_4_, 28 mM D-glucose, 0.1 mM acetosyringone, 4.1 mM L-Cys, 0.02% (w/v) Silwet L-77] to OD_600_ = 0.5, mixed with 1:1 (v:v), and poured into the root of soybean seedlings with 5 ml per plant. The treatment was performed three times, with an interval of 5 days each time. RT-qPCR was used to examine the silencing effect in the first leaves in soybean seedlings 3 weeks after initial *Agrobacterium tumefaciens* treatment before subjected to salt stress treatment.

### Hairy Roots Induction and Biomass Statistics

Hairy roots were prepared using *Agrobacterium rhizogenes*-mediated soybean cotyledon induction according to previously published protocols (Chen et al., 2014; An et al., 2017) with minor modification. After surface sterilization for 1 h using chlorine vapor, the soybean cultivar Mustang seeds were placed on germination medium (Gamborg’s B5 medium with 87.64 mM sucrose, 1.88 mM MES, and 8 g/L agar, pH 5.75) and cultivated at 25°C and 14/10 (L/D) photoperiod for 7 days. *Agrobacterium rhizogenes* strain K599 carrying the recombinant plasmid for *GmNHX5*-overexpression or knockout were used for soybean hairy root induction. Soybean seedlings were placed in a sterile petri dish, the cotyledons were cut off, and the cotyledons were soaked in a solution (87.64 mM sucrose, 1.88 mM MES, 0.2 mM acetosyringone, 1.66 mM L-cystine, and 1 mM DTT, pH 5.4) containing corresponding *Agrobacterium rhizogenes* strains at OD_600_ = 0.5 for 30 min, and cultured on sterile filter paper in the dark at 25°C for 5 days. The cotyledons were washed with sterile water and transferred to the induction medium (Gamborg’s B5 medium with 43.82 mM sucrose, 1.88 mM MES, 400 mg/L cefotaxime sodium, 100 mg/L carbenicillin disodium, 100 mg/L vancomycin hydrochloride, 100 mg/L timentin, and 8 g/L agar, pH 5.75) and incubated in the dark at 25°C for 15 days.

Hairy roots were cut off and used to detect the expression of *GmNHX5* (for OE hairy roots) or gene editing (for KO hairy roots), and about 1 cm of hairy roots were reserved for subsequent experiments. The DNA of the transformed hairy roots was amplified by GmNHX5KD-F/R and sequenced to confirm the knockout effect. The partially cut hairy roots were used for RT-qPCR analysis or sequencing, and the remaining hairy roots (about 1 cm in length) with their cotyledons were continued to be cultured in the induction medium containing 0, 70, and 150 mM NaCl for another 15 days before analysis. For hairy root biomass statistics, the maximum root length was measured as the highest length of hairy roots produced on each cotyledon. Fresh weight was measured as the weight of all hairy roots for each cotyledon.

### Generation of *GmNHX5*-Overexpressing Soybean Plants

Soybean plants overexpressing *GmNHX5* were generated using *Agrobacterium tumefaciens*- mediated cotyledonary node transformation following a previously published protocol (Paz et al., 2004; Lee et al., 2013), and the soybean cultivar Mustang was used as the transformation recipient. *Agrobacterium tumefaciens* strain EHA105 carrying plasmid for *GmNHX5*-overexpression was used for soybean transformation. The glyphosate-screened soybean seedlings were further verified using PCR (with primers BarS/X), colloidal gold test, and Southern blot analysis to confirm the transformation event.

### Colloidal Gold Test

The colloidal gold test was performed using colloidal gold test strips (QuickStix for PAT/bar, Envirologix) following the manufacturer’s protocol.

### Southern Blot

DNA isolated from *GmNHX5*-overexpressing soybean seedling leaves were used for Southern blot analysis. The digoxigenin-labeled probe was synthesized using primers BarS/X with a labeling kit (PCR DIG probe synthesis kit, Roche) following the manufacturer’s protocol. The DNA samples digested with *Hin*dIII, together with a molecular marker (DIG-labeled DNA molecular weight marker II, Roche), were separated on a 1% agarose gel, transferred to a positively charged nylon membrane, and subjected to UV cross-linking and probe hybridization. The labeled DNA fragments were developed using a DIG detection kit (DIG-High prime DNA labeling and detection starter kit I, Roche).

### Phenotyping and Physiological Analysis

Three-week-old WT and *GmNHX5*-overexpressing soybean seedlings were irrigated with Hoagland’s nutrient solution with 0 or 150 mM NaCl for 20 days, leaf dry weight and leaf fresh weight were measured as the total weight of fresh or kiln-dried leaves from single seedlings.

Soybean seedlings were exposed to 0 or 150 mM NaCl for 6 days before determination of free proline, betaine, and malondialdehyde (MDA). Free proline content was measured according to a previously published protocol (Bates et al., 1973). Betaine was extracted with ODS-C18 column and measured using an HPLC based method (Schmedes and Ga, 1989). The MDA content was determined according to a thiobarbituric acid (TBA) method (Schmedes and Ga, 1989).

### Determination of Na^+^ and K^+^ Ion Content

Three-weeks-old seedlings of *GmNHX5*-overexpressing T_3_ soybean lines #3 and #4, and untransformed soybean cultivar Mustang was used for Na^+^ and K^+^ content determination. Seedlings were exposed to 0 or 150 mM NaCl for 6 h before analysis.

Ion content determination was performed as previously described (Sun et al., 2019), with minor modification. The surface of the leaves, roots, and stems of materials post-treatment were washed with sterile water and were baked in an oven at 120°C for 30 min, and then dried at 80°C to a constant weight. The dried soybean material was ground into a fine powder, adding 10 mL anhydrous sulfuric acid, boiled at 170°C for 20 min, 220°C for 40 min, and 320°C for 2 h until the solution became clear. During the boiling process, a small amount of 30% hydrogen peroxide was added intermittently until a large amount of smoke appeared. After cooling, the product was diluted to 50 ml, and 100 μL of the diluted product was further diluted to 6 ml with distilled water. The diluted sample was filtered through a 0.45 μm membrane, and the content of K^+^ and Na^+^ ions was measured with an atomic absorption spectrophotometer (ZA3000, Hitachi).

### Accessions

Sequence information of genes involved in this study can be found in NCBI^2^ through following accessions. XM_006597582.2 (*GmNHX5*); NM_001258010.2 (*GmSOS1*); XM_003544313.4 (*GmSKOR*); XM_014764887.2 (*GmHKT1*); XM_003545450.3 (*GmAKT1*); XM_003553762.3 (*GmHAK5*); XM_026123971.1 (*GmNHX1*); XM_026124165.1 (*Actin*).

### Statistical Analysis

Statistical calculations were done with Microsoft Excel. Detailed statistical analysis is described in legend of each figure.

## Results

### *GmNHX5* Responds to Salt Stress

**FIGURE 1:**
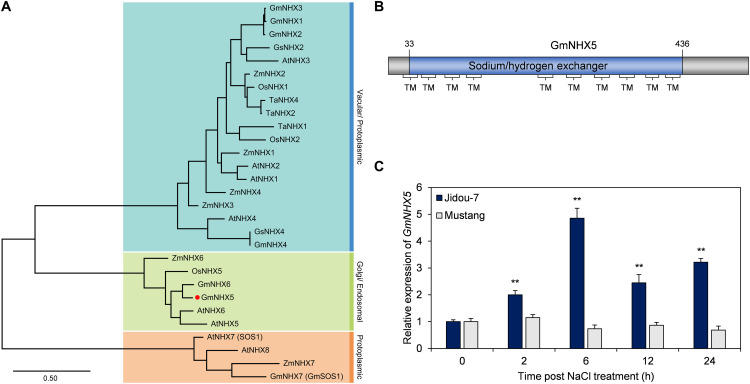
Characterization and expression of GmNHX5. **(A)** Phylogenetic analysis of GmNHX5 and its homologs from other plants. Protein sequences of NHXs from several plants were used for phylogenetic analysis using the maximum likelihood method and JTT matrix-based model. Species abbreviated as: Ta, *Triticum aestivum*; Os, *Oryza sativa*; Zm, *Zea may*s; Gm, *Glycine max*; Gs, *Glycine soja*; At, *Arabidopsis thaliana*. Red dot emphasizes GmNHX5 used in this study. Colored sections indicate potential subcellular localization of corresponding proteins retrieved from UniProtKB. The scale bar indicates the number of amino acid substitutions per site. **(B)** Structural domains of GmNHX5. The colored section indicates Na^+^/H^+^ exchanger functional domain, Braces indicate transmembrane (TM) domains. **(C)** Relative expression of *GmNHX5* under salt stress. RT-qPCR was used to detect the expression of *GmNHX5* in soybean Jidou-7 and Mustang at 200 mM NaCl for the corresponding time. *Actin* was used as a reference gene. Each value represents the mean ± SE of three biological replicates relative to 0 h. Asterisks above the bars indicate significant differences from the values measured in Mustang (*P* < 0.05 by Student’s *t*-test).

The *Arabidopsis nhx5 nhx6* double mutant shows high sensitivity to salt stress, indicating that Golgi-localized NHXs may be involved in the tolerance of salt stress in plants (Bassil et al., 2011a). Phylogenetic analysis showed that GmNHX5 shared high sequence identity with AtNHX5 and AtNHX6 (Figure 1A), suggesting that GmNHX5 may also be localized in the Golgi apparatus and is relevant to salt tolerance. GmNHX5 possesses a typical sodium/hydrogen exchanger domain and has 10 transmembrane domains (Figure 1B), which indicates that GmNHX5 is also a membrane protein and may have similar functions to other NHXs, namely regulating the steady-state of H^+^ and Na^+^ on both sides of the membrane.

We further examined the association between the transcription level of *GmNHX5* and salt tolerance. The abundance of *GmNHX5* mRNA in the salt-tolerant soybean cultivar Jidou-7 increased upon salt treatment and reached its peak at 6 h after treatment, then slightly decrease afterward, while the expression in the salt-sensitive cultivar Mustang showed no significant changes (Figure 1C), indicating that *GmNHX5* may be involved in salt tolerance in soybean.

### *GmNHX5* Actively Transcribes in New Leaves and Vascular Tissues Under Salt Stress

**FIGURE 2:**
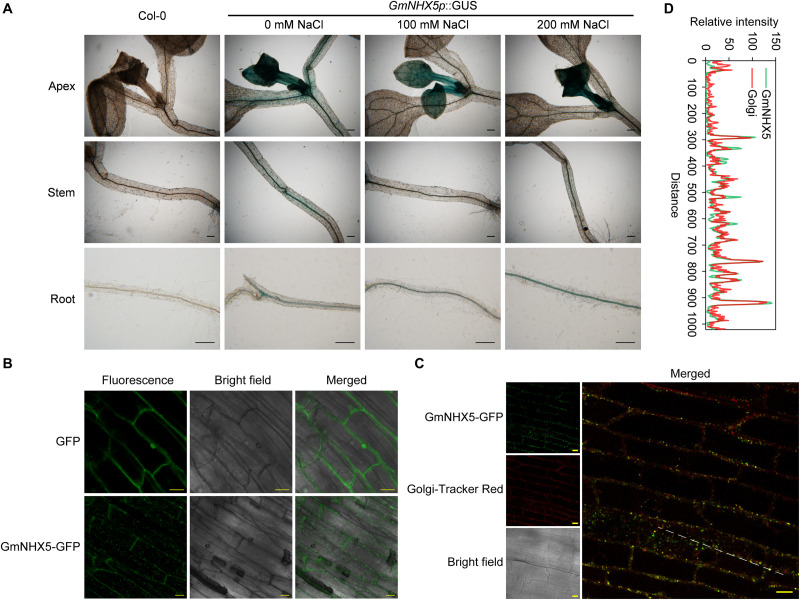
Detection of histological distribution of subcellular localization of GmNHX5. **(A)** Histochemical analysis of *GmNHX5* expression. GUS activity of *GmNHX5p*:GUS in response to salt stress in transformed *Arabidopsis* was detected by GUS staining. Five-day-old seedlings were treated with 0, 100, or 200 mM NaCl for 24 h before staining. **(B)** Fluorescence of GmNHX5-GFP or GFP in *Arabidopsis* roots. **(C)** Fluorescence of GmNHX5-GFP and its colocalization with fluorochrome Golgi-Tracker Red. Confocal imaging of the roots of 5-day-old *Arabidopsis* seedlings transformed with plasmids carrying *CaMV 35S*:GmNHX5-GFP or *CaMV 35S*:GFP expression loci. Representative images of three biological replicates are shown. Bars = 200 μm in panel **(A)**, Bars = 10 μm in panels **(B,C)**. **(D)** Line profile was used to illustrate colocalization between GmNHX5-GFP and Golgi-Tracker Red along the dotted line in panel **(C)**. Green and red lines indicate GmNHX5-GFP and Golgi-Tracker Red fluorescence profiles, respectively.

We used β-glucuronidase (GUS) as a reporter to assess *GmNHX5 promoter* activity in various tissues in *Arabidopsis*. The GUS activity driven by the *GmNHX5 promoter* was detected in new leaves at the stem apex, in the veins of mature leaves, and the vascular tissues of the stems and roots (Figure 2A). Upon salt treatment, the GUS activity in the new leaves increased, while decreased in the veins of the mature leaves and the vascular tissues of the stem, and the GUS activity in the vascular tissues of the root increased with the salt treatment. These results indicate that the transcription of *GmNHX5* is regulated by salt stress, and the *GmNHX5* gene is actively transcribed in new leaves and roots under salt stress. Given that the root system is the tissue where the plant directly contacts the soil solution, we speculate that the transcriptional up-regulation of *GmNHX5* in root vascular tissues and new leaves may lead to the up-regulation of GmNHX5 protein abundance in these tissues, thereby increases the adaptability to salt stress in roots, and protects young tissues from osmotic stress, respectively.

### GmNHX5 Localizes to the Golgi Apparatus

The subcellular localization of protein determines the possible way of its function. To reveal the subcellular localization of GmNHX5, we examined the roots of *Arabidopsis* expressing the GmNHX5-GFP fusion protein. Using the plants transformed with the plasmid that harbors GFP alone as a control, a granular-distributed fluorescence pattern was detected in cells expressing the GmNHX5-GFP fusion protein (Figure 2B). In order to confirm the possibility of GmNHX5 distribution in the Golgi apparatus, we introduced the fluorochrome Golgi-Tracker Red to label the cellular Golgi apparatus. Granular distribution of fluorescence was also observed in cells labeled with Golgi-Tracker Red, and could co-localize with most of the fluorescence of GmNHX5-GFP (Figure 2C), indicating that GmNHX5 was distributed in the Golgi apparatus. The co-localization efficiency was further demonstrated by line profile (Figure 2D). These results indicate that GmNHX5 was distributed in the Golgi apparatus intracellularly.

### Silencing of *GmNHX5* Reduces Salt Tolerance in Soybean

**FIGURE 3:**
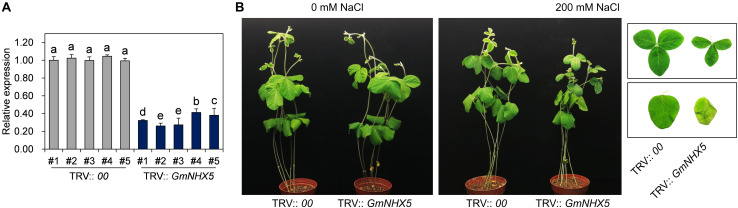
Response of *GmNHX5*-silencing soybean plants to salt stress. **(A)** Silencing efficiency of *GmNHX5* in TRV-inoculated plants. The relative expression of *GmNHX5* was detected using RT-qPCR, and normalized to the values measured in TRV: *00*-#1. *Actin* was used as a reference gene. Each value represents the mean ± SE of three technical replicates. Different letters above the bars indicate significant differences (*P* < 0.05 by one-way ANOVA and Duncan’s multiple comparison). **(B)** Phenotype of *GmNHX5*-silencing plants under salt stress condition. TRV-inoculated soybean plants were subjected to 0- or 200 mM NaCl solution treatment for 5 days. The images on the right show the phenotype of simple (top) and ternately compound leaves (bottom) of indicated plants under salt stress.

As a preliminary exploration of the potential function of *GmNHX5* involved in plant salt tolerance, we employed TRV-mediated VIGS technology and observed the tolerance of silent plants to salt stress. TRV carrying a specific segment of *GmNHX5* mRNA produced *GmNHX5* gene silencing with an efficiency of over 50% in TRV-inoculated Jidou-7 plants (Figure 3A). The silencing of *GmNHX5* did not significantly affect plant development (Figure 3B). After 200 mM salt treatment, *GmNHX5*-silencing soybean plants showed wilting at the whole plant level and obstructed apical development, simple leaves showed symptoms of chlorosis and wilting, ternately compound leaves showed obvious growth retardation, while the growth status of the control (TRV: *00*) plants was significantly better, and less affected by salt stress (Figure 3B). These results indicate that the silencing of *GmNHX5* reduces the adaptability of soybean to salt stress.

### *GmNHX5* Positively Regulates Salt Tolerance in Soybean Hairy Roots

**FIGURE 4:**
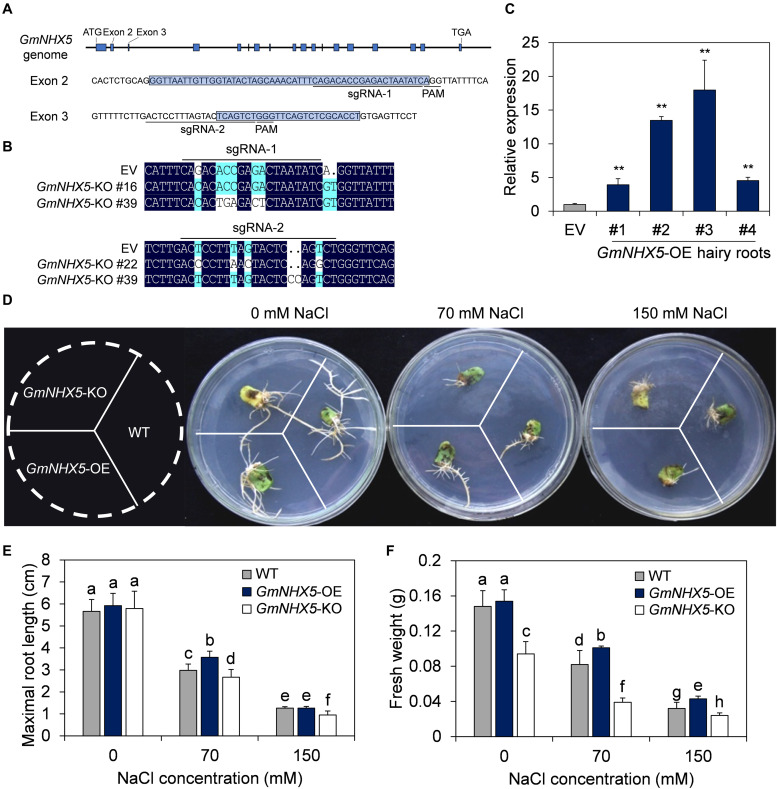
Functional characterization of *GmNHX5* in soybean hair roots. **(A)** Schematic diagram of the of sgRNA binding sites used for CRISPR/Cas9-mediated knockout of *GmNHX5* on the genome. The start codon (ATG), Exons (blue box), stop codon (TGA), sequences of Exon 2 and Exon 3 and their flanking sequences are indicated, sgRNAs and PAMs are underlined. **(B)** Sequence alignment of the knockout (KO) hairy roots at the sgRNA1 and sgRNA2 positions. EV, hairy roots transformed with empty vector pBSE401. **(C)** Expression of *GmNHX5* in *GmNHX5*-overexpressing (OE) hairy roots. The relative expression of *GmNHX5* was detected using RT-qPCR, and normalized to the values measured in the hair roots transformed with empty vector pCAMBIA3301 (EV). *Actin* was used as a reference gene. Each value represents the mean ± SE of three technical replicates of one hairy root induced by a single cotyledon. Asterisks above the bars indicate significant differences from the values measured in EV (*P* < 0.05 by Student’s *t*-test). **(D)** Phenotype of *GmNHX5*-overexpressing and knockout hairy roots under salt stress. Hairy roots were cultured on 1/4 MS medium with 0, 70, and 150 mM NaCl for 15 days before observation. WT, hairy roots induced by *Agrobacterium rhizogenes* without plasmid. Representative images of three biological replicates are shown. Maximum length **(E)** and fresh weight **(F)** of soybean hairy roots in **(D)**. The data in panels **(E,F)** shows the mean ± SD of 30 independent measurements. Different letters indicate significant difference (*P* < 0.05 by one-way ANOVA/Duncan).

We next analyzed the function of *GmNHX5* on salt tolerance in hairy roots induced by soybean cotyledons. The plasmid carrying the *GmNHX5* open reading frame (ORF) driven by the *CaMV 35S* promoter was used for overexpression (OE), and the CRISPR/Cas9 technique was used to conduct *GmNHX5* targeted knockout (KO). We specifically designed sgRNA-1 and sgRNA-2 that target two *GmNHX5*-specific sites to increase knockout efficiency (Figure 4A). After sequencing, we found that *GmNHX5* in soybean hairy roots transformed with the plasmid expressing Cas9 and sgRNAs had frameshift mutations (Figure 4B), which will result in the malfunctional GmNHX5 to be expressed. Meanwhile, the relative expression of *GmNHX5* in the OE hairy roots was significantly higher than that in the hairy roots transformed with the empty vector (EV) (Figure 4C).

The growth status and maximum root length of OE and KO hairy roots were similar on medium free of NaCl (Figures 4D,E), whereas the fresh weight of the OE and the wild-type (WT) were significantly higher than the KO (Figure 4F). The difference in fresh weight (Figure 4F) in 0 mM NaCl treatment between the WT-, OE-, and KO hairy roots suggest a possible K^+^ transport role of GmNHX5 in soybean, and the reduction in fresh weight in *GmNHX5*-KO hairy roots may be due to impaired K^+^-uptake and utilization where *GmNHX5* was knocked out. On the medium containing 70 mM NaCl, the growth status of the OE hairy roots was superior to the others, the maximum root length and fresh weight of the OE were significantly higher than that of the KO and WT hairy roots. When the NaCl concentration increased to 150 mM, the maximum root length of the OE was not significantly different from the WT, but the fresh weight was significantly higher than that of the WT and the KO. These results indicate that *GmNHX5* positively regulates salt tolerance in soybean hairy roots.

### *GmNHX5* Enhances the Adaptability to Salt Stress in Transformed Soybean

**FIGURE 5:**
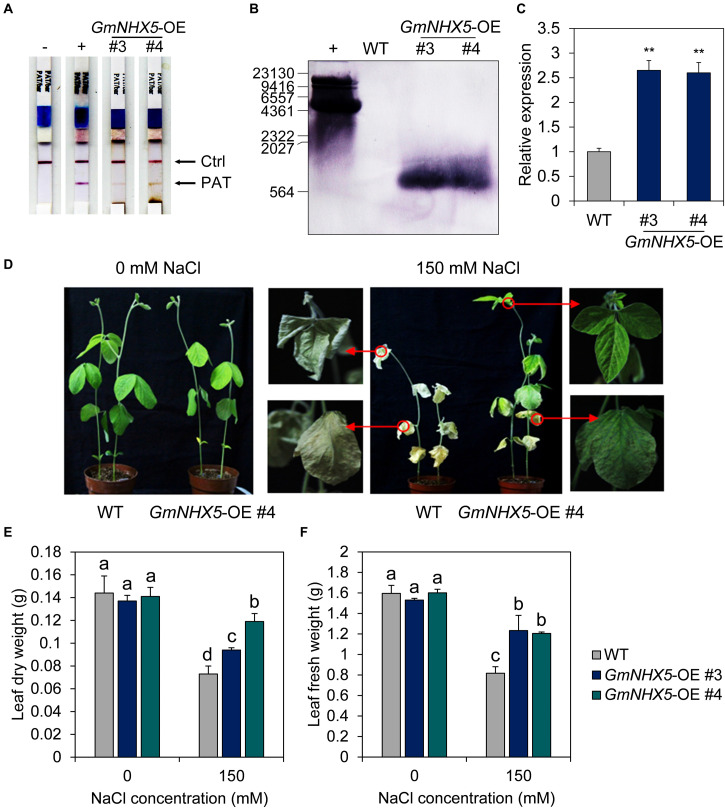
Overexpression of *GmNHX5* enhances salt tolerance in soybean. **(A)** Identification of *GmNHX5-*overexpressing soybean plants. Leaf extract from independent *GmNHX5*-OE transformed T_3_ lines #3 and #4 were tested using colloidal gold strips. Phosphinothricin acetyl-transferase (PAT) refers to the herbicide resistance marker on the plasmid. –, untransformed soybean; +, positive control. **(B)** Southern blot analysis of the copy number of plasmids in the genome of *GmNHX5-*overexpressing plants. +, plasmids for soybean transformation. Numbers indicate molecular weight standards. **(C)** Expression of *GmNHX5* in *GmNHX5*-overexpressing (OE) soybean plants. The relative expression of *GmNHX5* was detected using RT-qPCR, and normalized to the values measured in the untransformed soybean (WT). *Actin* was used as a reference gene. Each value represents the mean ± SE of three biological replicates. Asterisks above the bars indicate significant differences from the values measured in WT (*P* < 0.05 by Student’s *t*-test). **(D)** Phenotypes of soybean plants overexpressing *GmNHX5* under salt stress. Three-weeks-old WT and *GmNHX5-*overexpressing soybean seedlings were irrigated with Hoagland’s nutrient solution with 0 or 150 mM NaCl for 20 days. Arrows indicate partial close-ups of the plant. Leaf dry weight **(E)** and leaf fresh weight **(F)** were measured as the total leaf weight in independent *GmNHX5*-OE transformed T_3_ lines #3 and #4. The data in panels **(E,F)** shows the mean ± SD of triplicate measurements. Different letters indicate significant difference (*P* < 0.05 by one-way ANOVA/Duncan).

The fact that *GmNHX5* positively regulates salt tolerance in hairy roots suggests that the gene may functions similarly at the organism level. To test this hypothesis, we generated *GmNHX5*-overexpressing plants in the salt-sensitive variety Mustang. Colloidal gold test on the phosphinothricin acetyl-transferase (PAT), a selective marker on the plasmid, showed that the protein was detected in #3 and #4, two transformed T_3_ soybean lines (Figure 5A). Southern blot analysis showed that the plasmid T-DNA carrying the *CaMV 35S*: *GmNHX5* expression cassette was integrated into the soybean genome in a single copy (Figure 5B). The relative transcription level of *GmNHX5* in #3 and #4 was significantly higher than that in the WT (Figure 5C). Under conventional conditions, the growth of *GmNHX5*-OE plants was not significantly different from that of WT. After treatment with 150 mM NaCl for 20 days, WT plants showed strong wilting, chlorosis, and growth obstacles, whereas the condition of *GmNHX5*-OE plants was significantly better, with a merely mild wilting phenotype (Figure 5D). Furthermore, biomass analysis showed that under conventional conditions, the leaf dry weight and leaf fresh weight of *GmNHX5*-OE plants were not significantly different from those of WT plants (Figures 5E,F); under salt stress, the leaf dry weight and leaf fresh weight of *GmNHX5*-OE plants were markedly higher than those of WT plants, which was consistent with the observed phenotype.

**FIGURE 6:**
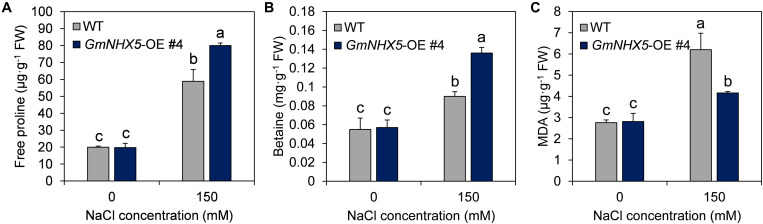
Physiological response of *GmNHX5*-overexpressing soybean to salt stress. Content of free proline **(A)**, betaine **(B)**, and MDA **(C)** were measured in indicated *GmNHX5*-overexpressing T_3_ lines 6-days after 0 or 150 mM NaCl treatment. The data shows the mean ± SD of triplicate measurements. Different letters indicate significant difference (*P* < 0.05 by one-way ANOVA/Duncan).

An important mechanism by which plants respond to and adapt to salt stress is the accumulation of osmotic adjustment substances, such as free proline and betaine. Under normal conditions, the contents of free proline (Figure 6A) and betaine (Figure 6B) in *GmNHX5*-OE plants were not significantly different from those in WT plants. However, when exposed to salt stress, the increase in the content of these two osmotic adjustment substances in *GmNHX5*-OE plants was much greater than that in WT plants. Meanwhile, *GmNHX5*-OE plants accumulated less MDA than WT plants under salt stress (Figure 6C), indicating that GmNHX5 attenuated lipid peroxidation caused by salt stress-induced ROS.

### *GmNHX5*-Overexpressing Soybean Has Higher K^+^/Na^+^ Ratio Under Salt Stress

In addition to accumulating osmotic adjustment substances, the transportation and compartmentalization of K^+^ and Na^+^ at the cellular and histological levels are also one of the main manners for plants to detoxify salt stress (Qiu, 2016).

**FIGURE 7:**
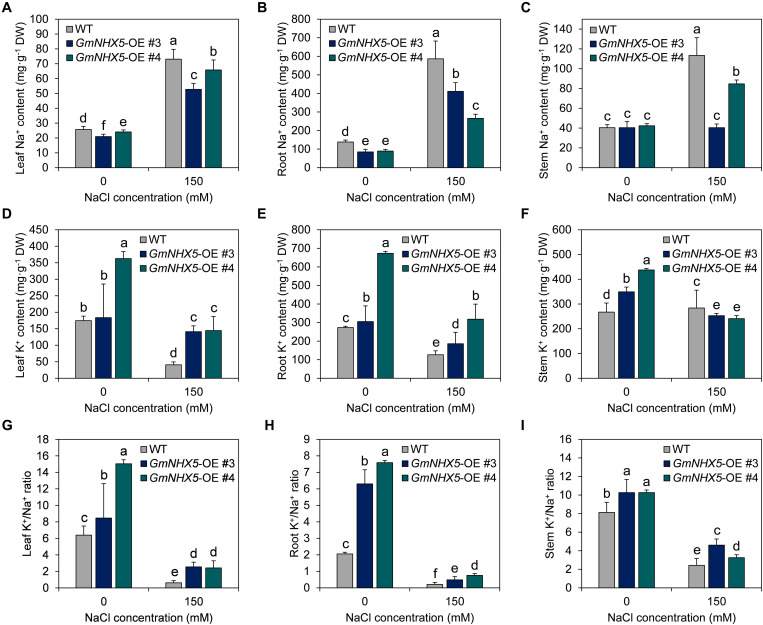
Na^+^, K^+^, and K^+^/Na^+^ ratio in soybean overexpressing *GmNHX5*. Content of Na^+^ **(A–C)** and K^+^ **(D–F)** was measured in #3 and #4, two independent *GmNHX5*-overexpressing soybean T_3_ lines 6 h after 0 or 150 mM NaCl treatment. Leaves, roots, and stems were examined to show the histologic distribution of Na^+^ and K^+^. K^+^/Na^+^ ratio was separately calculated in leaves **(G)**, roots **(H)**, and stems **(I)**. The data shows the mean ± SD of triplicate measurements. Different letters indicate significant difference (*P* < 0.05 by one-way ANOVA/Duncan).

In order to inquire into the mechanism and characteristics of *GmNHX5*-mediated salt tolerance in plants, we examined the content of K^+^ and Na^+^ in #3 and #4, two independent *GmNHX5*-OE T_3_ lines. Under normal conditions, the content of Na^+^ in OE leaves was lower than that of WT leaves (Figure 7A); the Na^+^ content in OE roots was also lower than that in WT plants (Figure 7B), while the overexpression of *GmNHX5* did not affect the Na^+^ content in stems (Figure 7C). In addition, under normal conditions, the K^+^ content of OE plants was higher than that of WT plants in all tissues tested (Figures 7D–F). When exposed to salt stress, Na^+^ content in all tissues detected in OE plants was significantly lower than that in WT plants, including leaves (Figure 7A), roots (Figure 7B), and stems (Figure 7C). The content of K^+^ in leaves (Figure 7D) and roots (Figure 7E) of OE plants were significantly higher than that of WT plants, and the content of K^+^ in stems of OE plants was lower than that of WT plants except (Figure 7F).

As a result, under normal conditions, the K^+^/Na^+^ ratio of *GmNHX5*-OE plants was higher than that of WT plants (Figures 7G–I). Under salt stress, the K^+^/Na^+^ ratio in leaves (Figure 7G), roots (Figure 7H), and stems (Figure 7I) of OE plants was also higher than that of WT plants. The higher K^+^/Na^+^ ratio of OE plants under salt stress may be the reason why they were more accommodating to salt stress.

### *GmNHX5* Regulates Salt-Responsive Genes

**FIGURE 8:**
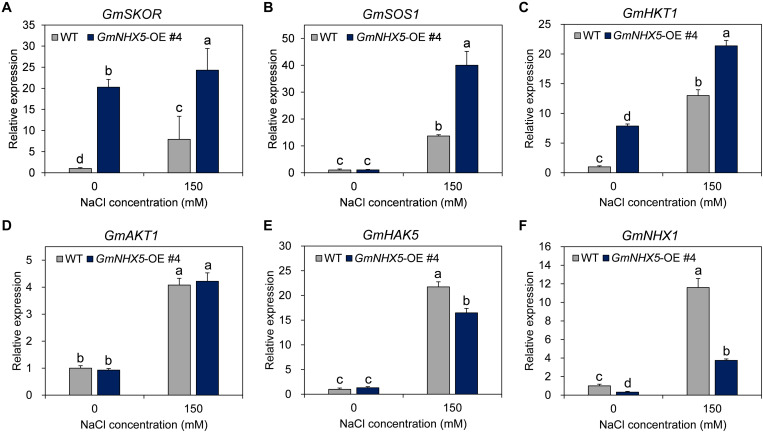
Expression analysis of salt tolerance related genes. The relative transcription level of *GmSKOR* **(A)**, *GmSOS1* **(B)**, *GmHKT1* **(C)**, *GmAKT1* **(D)**, *GmHAK5* **(E)**, and *GmNHX1* **(F)** were detected in the *GmNHX5*-overexpressing T_3_ soybean seedlings 6 h after 0 or 150 mM NaCl treatment, and normalized to the values measured in WT exposed to 0 mM NaCl. *Actin* was used as a reference gene. Each value represents the mean ± SE of three biological replicates. Different letters indicate significant difference (*P* < 0.05 by one-way ANOVA/Duncan).

Our results suggested that GmNHX5 localized in the Golgi apparatus and regulated the accumulation of Na^+^ and K^+^, which implies that it may indirectly affect the uptake of these two ions. Transcriptional regulation of salt-related genes is a vital way for plants to strengthen their adaptability under salt stress. In order to reveal the regulatory effect of *GmNHX5* on salt-responsive genes, we examined the transcriptional expression of several salt-responsive genes (Figures 8A–F), including K^+^ channel *GmSKOR* (Figure 8A), *GmHKT1* (Figure 8C), and *GmAKT1* (Figure 8D); K^+^ pump *GmHAK5* (Figure 8E); and exchangers *GmSOS1* (Figure 8B) and *GmNHX1* (Figure 8F) in soybean overexpressing *GmNHX5*. It was found that under normal conditions, overexpression of *GmNHX5* induced the expression of *GmSKOR* and *GmHKT1*; reduced the expression of *GmNHX1*; but did not affect the expression of *GmSOS1*, *GmAKT1*, and *GmHAK5*. Under salt stress, however, the overexpression of *GmNHX5* increased the expression of *GmSKOR*, *GmSOS1*, and *GmHKT1*; decreased the expression of *GmHAK5* and *GmNHX1*; and did not affect the expression of *GmAKT1*. These results indicate that the overexpression of *GmNHX5* affects the expression level of salt-related genes, but the specific molecular mechanism by which GmNHX5 regulates the expression of these genes remains to be further studied.

## Discussion

Given that NHXs are ion/proton exchangers that directly regulate cellular Na^+^ or K^+^ uptake or compartmentalization, such genes have been shown to possess the capability to improve the salt tolerance in various plant species, including *Medicago sativa* (Zhang et al., 2015), *Capsicum annuum* (Bulle et al., 2016), *Cucumis sativus* (Wang et al., 2013), etc. The key to NHX-mediated increase in plant salt tolerance is to increase the ratio of cytoplasmic K^+^/Na^+^ to avoid excessive accumulation of these two ions, so as to ultimately reduce the damage to organelles. Although many studies have shown that members of the NHX family of plants have the ability to exchange Na^+^ (Shi et al., 2002), there is also evidence that they may function by transporting K^+^ (Bassil et al., 2011b; Wang L. et al., 2015). In view of the large number of ion channels and ion pumps in the membrane system of plant cells, there is currently no direct evidence of the exact ion molecule to be transported by NHXs. Although there are still in debate over the actual ions used by NHXs to exchange H^+^, it is certain that most of the NHXs has been discovered reduces the Na^+^ content in cytoplasm or maintain high K^+^/Na^+^ ratio.

According to the structural characteristics, NHXs are mainly classified into two categories, AtNHX1, AtNHX2, AtNHX3, AtNHX4, AtNHX7/SOS1, and AtNHX8 are the first type of NHX, which are located in the plasma membrane and vacuolar membrane. The main functional domain of these NHXs belongs to the “Na^+^-H^+^_Exchanger” superfamily and was considered to have an important role in maintaining the Na^+^ or K^+^ (Bassil et al., 2011b) levels, Na^+^/K^+^ homeostasis and pH stability (Dibrov and Fliegel, 1998; Rodríguez-Rosales et al., 2009). AtNHX5 and AtNHX6 belong to the second type of NHX, which is located on the vesicle membrane in the endoplasmic reticulum and the Golgi network, whose main domain belongs to the “MFS” superfamily, and was found to regulate pH and affects the sorting behavior of the Golgi network (Bassil et al., 2011a; Reguera et al., 2015; Date et al., 2016). According to phylogenetic analysis, GmNHX5 was highly similar to NHXs localized by the Golgi network/endosomal system represented by AtNHX5 and AtNHX6 (Figure 1A). The results of subcellular localization further indicate that GmNHX5 was localized on the Golgi network (Figure 2C), in accordance with the distribution pattern of AtNHX5 (Bassil et al., 2011a). The Golgi network is considered to be a center for cell component transportation, which connects to the endoplasmic reticulum, vacuole, and plasma membrane (Larkin et al., 2016). Under salt stress conditions, plants produce large amounts of secondary metabolites and discharge or isolate salt ions that destroy intracellular structures, which all depend on the role of the Golgi network (Ueda et al., 2016). Thereby, we speculate that the salt tolerance-related molecular mechanism of GmNHX5 may rely on the function of the Golgi network. In addition to the distribution within the cell, the histological distribution of the protein also plays a decisive role in its function. GUS activity driven by *OsNHX1 promoter* was localized to the guard cells and trichome, whereas *OsNHX5 promoter*-GUS activity was localized to the root tip and pollen grains (Fukuda et al., 2011). In this study, it was found that the *GmNHX5* promoter activity was distributed in the young leaves and roots in the transformed *Arabidopsis*, and with the increase of the intensity of salt stress, the distribution of GUS signal enriched in the young leaves and the growing point, and the vascular bundles in the roots. Given that the Golgi apparatus connects vacuole and plasma membrane, the localization of GmNHX5 on the Golgi apparatus (Figure 2C) of the vascular (Figure 2A) indicating that it may participate in the transport of K^+^. Since the K^+^ content in the root system is higher than that in the stem and leaves under normal condition (Figures 7D–F), we speculate that GmNHX5 may participate in the transfer of K^+^ absorbed by the root to the aboveground organs to protect the young tissues, and at the same time regulate the utilization of K^+^.

TRV-VIGS-induced *GmNHX5* silencing plants showed enhanced sensitivity to salt stress, which gave us a glimpse of the functions of GmNHX5 related to salt tolerance and prompted us to further explore the functions of GmNHX5 in diverse ways. The hairy roots of plants induced by *Agrobacterium rhizogenes* inherit the plasmid carried in the bacteria, and are considered to be an ideal material for genetic function verification at the histological level. Due to its fast and convenient characteristics, hairy roots have been used in the study of physiological mechanisms in various plant species, including *Solanum lycopersicum* (Ron et al., 2014), *Saussurea involucrate* (Li et al., 2006), and *Glycine max* (Kim et al., 2009). In this study, *GmNHX5* was overexpressed or knocked out with CRISPR/Cas9 in soybean hairy roots, and it was found that hair roots overexpressing *GmNHX5* had lower salt sensitivity, which was opposite to that of *GmNHX5* was knocked out (Figure 4). In addition, in view of the significant increase of K^+^ content in the roots of *GmNHX5*-OE plants (Figure 7E), the decrease in the biomass of *GmNHX5*-KO hairy roots may also be related to the impaired of K^+^ uptake. These results are consistent with the function of *AtNHX5* in *Arabidopsis* (Zhu et al., 2018) and prove that the expression of *GmNHX5* was positively correlated with the salt tolerance of soybean. In addition to the hairy root experiments, this study also generated the T_3_ generation transformed lines overexpressing *GmNHX5* on the soybean Mustang, and verified the transformation event from the protein level of the reporter gene PAT (bar), the relative transcription level of *GmNHX5*, and the presence of plasmid integration in the soybean genome (Figure 5). We also examined the influence of *GmNHX5* on the physiological state of soybean plants from the contents of proline, betaine, and MDA (Figure 6). As well-known osmotic adjustment substances in plants, the content of free proline and betaine increases significantly when undergoing osmotic stress. Varieties with higher drought and salt tolerance tend to accumulate more free proline, it can be considered that the free proline content in plants reflects the osmotic tolerance to some extent (Trinchant et al., 2004; Nedjimi, 2013; Al-Farsi et al., 2020). In this study, we found that *GmNHX5-*overexpressing soybean plants accumulated more free proline and betaine when subjected to salt stress, but the contents of these two osmotic adjustment substances were not significantly different from WT under normal conditions, indicating that *GmNHX5* does not directly increase the background levels of proline and betaine, but may regulate their accumulation in a salt stress-dependent manner. MDA is a product of oxidative damage to the cell membrane, and its accumulation level indicates the degree of cell damage (Wang, 2014). The MDA content of *GmNHX5*-overexpressing plants under salt stress was significantly lower than that in WT plants, indicating that *GmNHX5* may protect cells from oxidative damage.

Na^+^, K^+^, and Cl^–^ are the main substances that affect the osmotic pressure of plant cells. Whether in monocotyledonous or dicotyledonous plants, the presence of these ions constitutes 80–95% of the osmotic pressure (Hastings and Gutknecht, 1974; Adams et al., 1992). Our results suggest that GmNHX5 positively regulates soybean tolerance to salt stress, we speculate that this may be achieved by multiple ways: On the one hand, GmNHX5 may regulate the increase of K^+^/Na^+^ ratio (Figures 7G–I); On the other hand, it may also increase the content of osmotic adjustment substances. In view of the similarity of the electrochemical characteristics of Na^+^ and K^+^, we conjecture that the transport activity of GmNHX5 is suitable for both of these two ions. As a homolog of AtNHX6 which participates in the maturation process of the pre-vacuolar compartment and uptakes K^+^ into the Golgi network (Bassil et al., 2011a; Wang L. et al., 2015; Zhu et al., 2018), we speculate that GmNHX5 may function in the following ways: (1) Transporting Na^+^ to the *trans*-Golgi network and pre-vacuolar compartment to carry out the secretion of Na^+^ directly; (2) Transporting K^+^ in the cytoplasm to the Golgi apparatus, leading to acidification of the cytoplasm and activation of intracellular signals such as cytosolic pH. These signals may further act on transcription factors in the nucleus and regulate the expression of salt-responsive genes such as *GmSKOR*, *GmSOS1*, *GmHKT1*, and *GmNHX1*, and induce the contents of free proline and betaine, to combat ROS burst and organelle damage caused by salt stress. Since ROS induces the transcription of *HAK5* (Huang et al., 2019), the reduced *GmHAK5* expression in the *GmNHX5*-OE plants (Figure 8E) may be the mobilization of ROS-scavenging system. The heterologous expression of *GmNHX1* can positively regulate the Na^+^ flow rate of *Arabidopsis* roots under salt stress, and enhance salt tolerance (Sun et al., 2019). Our results showed that the overexpression of GmNHX5 constitutively down-regulated the expression of *GmNHX1* (Figure 8F). It is speculated that the regulation of cytoplasmic K^+^/Na^+^ balance by GmNHX5 reduces the requirement for the expression of the vacuole-localized GmNHX1, and GmNHX5 may play upstream of GmNHX1 in conferring salt tolerance. The induced expression of *GmSOS1* (Figure 8B) in *GmNHX5*-OE plants may also be one of the reasons for the reduced intracellular Na^+^. The expression of *AKT1* gene was decreased in the salt-tolerant cultivar of *Fragaria* variety under salt stress condition (Garriga et al., 2017), but we found that the expression of *GmAKT1* was unchanged by salt stress, which may be due to the differences in species and salt tolerance mechanisms. The specific molecular mechanism of *GmNHX5* in regulating the expression of these salt-responsive gene remains to be further studied. These results not only deepen our understanding of the role and molecular mechanism of NHXs located in the Golgi apparatus in response to salt stress, but also provide genetic resources for the cultivation of salt-tolerant plant materials.

## Data Availability Statement

The original contributions presented in the study are included in the article/Supplementary Material, further inquiries can be directed to the corresponding author/s.

## Author Contributions

DW, JC, and JZ supervised the project. DW and JZ conceived and designed the experiments. TS, NM, and CW performed the experiments and analyzed the data. HF and MW contributed reagents, materials, and analysis tools. TS and NM wrote the manuscript. All authors contributed to the article and approved the submitted version.

## Conflict of Interest

The authors declare that the research was conducted in the absence of any commercial or financial relationships that could be construed as a potential conflict of interest.

## Footnotes

1 https://fiji.sc/Fiji

2 https://www.ncbi.nlm.nih.gov/
