# Supplementary material for: A Golgi-Localized Sodium/Hydrogen Exchanger Positively Regulates Salt Tolerance by Maintaining Higher K+/Na+ Ratio in Soybean
Source: Front Plant Sci. 2021 Mar 9;12:638340. doi: 10.3389/fpls.2021.638340 (PMC7985447; doi:10.3389/fpls.2021.638340)
Supplement: Supplementary file 1 [file Table_1.DOCX]

**Table S1** Primer information

| Primer name | Sequence (5’→3’) | Amplification length (bp) | Application |
| --- | --- | --- | --- |
| 1381-GmNHX5p-F | GAATTCATTTACGTGATGATTCAATGGTAA | 2012 | Construction of plasmid for *GmNHX5* histological localization analysis. |
| 1381-GmNHX5p-R | AAGCTTTATTGTTCTCCTTGCCGGAAGCAA |  |  |
| GmNHX5GFP-F | AGATCTGATGGCCTCGGAATTAGA | 1616 | Construction of plasmid for *GmNHX5* subcellular localization. |
| GmNHX5GFP-R | GGTAACCTGATGAATAGTGGTTCTGGCCAT |  |  |
| GmNHX5VIGS-F | GAATTCGGAACTGGGCACAGATGTCA | 309 | Construction of plasmid to generate *GmNHX5*-silenced plants. |
| GmNHX5VIGS-R | CTCGAGGCCTTCAGCAAGCATGTACG |  |  |
| GmNHX5OE-F | AGATCTGATGGCCTCGGAATTAGAA | 1619 | Construction of plasmid for *GmNHX5* overexpression. |
| GmNHX5OE-R | GGTAACCTCATGATGAATAGTGGTTC |  |  |
| GmNHX5-DT1-BsF | ATATATGGTCTCGATTGAGACACCGAGACTAATATCGTT | 726 | Construction of plasmid for *GmNHX5* knockout. |
| GmNHX5-DT1-F0 | TGAGACACCGAGACTAATATCGTTTTAGAGCTAGAAATAGC |  |  |
| GmNHX5-DT2-R0 | AACAGACTGAGTACTAAAGGAGCAATCTCTTAGTCGACTCTAC |  |  |
| GmNHX5-DT2-BsR | ATTATTGGTCTCGAAACAGACTGAGTACTAAAGGAGC |  |  |
| GmNHX5KD-F | CCTGCAGATCATGATGTTGGTCTTG | 580 | Amplification of the fragment used for sequencing to verify *GmNHX5* knockout in hairy roots. |
| GmNHX5KD-F | AAAGGCCAGTCCATATAAACAAAGT |  |  |
| BarS | TCAAATCTCGGTGACGGGC | 488 | Verification for T-DNA insertion in transformed plants/ hairy roots in *GmNHX5* overexpression or knockout experiments. |
| BarX | GCACCATCGTCAACCACTAC |  |  |
| qRT-GmNHX5-F | GTCTGGGTTCAGTCTCGCAC | 135 | RT-qPCR analysis of the expression of *GmNHX5* (XM_006597582.2). |
| qRT-GmNHX5-R | ATCAGAAAGAGCAAGCCACCA |  |  |
| qRT-GmSOS1-F | TTGTGCTGCATTTCTTCGAG | 120 | RT-qPCR analysis of the expression of *GmSOS1* (NM_001258010.2). |
| qRT-GmSOS1-R | CGTGCTTCTTCTCCTTCCAC |  |  |
| qRT-GmSKOR-F | GTGCCTTGTAACGTGTTGTGC | 210 | RT-qPCR analysis of the expression of *GmSKOR* (XM_003544313.4). |
| qRT-GmSKOR-R | CTGAGCTTTGACGTGTGCCA |  |  |
| qRT-GmHKT1-F | GGAATACTCGCACTTGCTTAAG | 129 | RT-qPCR analysis of the expression of *GmHKT1* (XM_014764887.2). |
| qRT-GmHKT1-R | GAGCAAAGCATCACAAACTGTA |  |  |
| qRT-GmAKT1-F | AGATGGCAGCCAATATAGTCTC | 247 | RT-qPCR analysis of the expression of *GmAKT1* (XM_003545450.3). |
| qRT-GmAKT1-R | CCCATTTACAACATTGTCGGTT |  |  |
| qRT-GmHAK5-F | AGAGTGCCCGGAATAGGACT | 122 | RT-qPCR analysis of the expression of *GmHAK5* (XM_003553762.3). |
| qRT-GmHAK5-R | GCCTTGATGGAGACAAACACA |  |  |
| qRT-GmNHX1-F | ACTGCGAAGCAATGCAATCA | 392 | RT-qPCR analysis of the expression of *GmNHX1* (XM_026123971.1). |
| qRT-GmNHX1-R | GGCCATTACGTTCAGTTGGTG |  |  |
| qRT-GmAct11-F | ATTTTGACTGAGCGTGGTTATTCC | 126 | RT-qPCR analysis of the expression of *Actin* (XM_026124165.1). |
| qRT-GmAct11-R | GCTGGTCCTGGCTGTCTCC |  |  |

Underlined sequences indicate restriction enzyme recognition sites.
