# Supplementary material for: A Golgi-Localized Sodium/Hydrogen Exchanger Positively Regulates Salt Tolerance by Maintaining Higher K+/Na+ Ratio in Soybean
Source: Front Plant Sci. 2021 Mar 9;12:638340. doi: 10.3389/fpls.2021.638340 (PMC7985447; doi:10.3389/fpls.2021.638340)
Supplement: Supplementary file 2 [file Table_2.DOCX]

**Table S2** Accessions of protein sequences used for phylogenetic analysis

| Annotation | UniProtKB accession | Annotation | UniProtKB accession |
| --- | --- | --- | --- |
| GmNHX5 | I1MFX0 | GmNHX1 | Q0IJ87 |
| GmNHX2 | F2X1X4 | GmNHX3 | Q330L2 |
| GmNHX4 | K7LA97 | GmNHX6 | A0A0R0JF15 |
| GmNHX7/GmSOS1 | H9CDQ2 | GsNHX2 | A0A445G0Q3 |
| GsNHX4 | A0A445JN25 | AtNHX1 | Q68KI4 |
| AtNHX2 | Q56XP4 | AtNHX3 | Q84WG1 |
| AtNHX4 | Q8S397 | AtNHX5 | Q8S396 |
| AtNHX6 | Q8RWU6 | AtNHX7/SOS1 | Q9LKW9 |
| AtNHX8 | Q3YL57 | TaNHX1 | Q94BM4 |
| TaNHX2 | Q94BM3 | TaNHX4 | A0A5J6DXT0 |
| OsNHX1 | A0A089PVR8 | OsNHX2 | Q2R0E9 |
| OsNHX5 | Q0J2X1 | ZmNHX1 | Q84MI0 |
| ZmNHX2 | A0A3L6DWM9 | ZmNHX3 | Q7XYX3 |
| ZmNHX4 | Q7XYX2 | ZmNHX6 | A0A3L6E1E2 |
| ZmNHX7 | A0A317Y7L3 |  |  |
